# Dynamic Encoding of Reward Prediction Error Signals in the Pigeon Ventral Tegmental Area during Reinforcement Learning

**DOI:** 10.1523/ENEURO.0355-25.2026

**Published:** 2026-03-03

**Authors:** Zhigang Shang, Jiashuo Zhang, Mengmeng Li, Suchen Li, Yinghui Wang, Lifang Yang

**Affiliations:** ^1^School of Electrical and Information Engineering, Zhengzhou University, Zhengzhou 450001, China; ^2^Henan Key Laboratory of Brain Science and Brain-Computer Interface Technology, Zhengzhou 450001, China; ^3^The Affiliated Encephalopathy Hospital of Zhengzhou University, Zhumadian 463000, China

**Keywords:** dynamic encoding, pigeon, reward prediction error, spike, ventral tegmental area

## Abstract

Reward prediction errors (RPEs) guide learning by comparing expected and obtained outcomes. In mammals, ventral tegmental area (VTA) activity is closely linked to RPE-like signaling, yet how avian VTA dynamics evolve during reinforcement learning remains less well characterized. Here we recorded VTA spiking in pigeons (two females and one male) performing a cue-guided operant task in which a green cue (cue^+^) predicted reward contingent on a key peck, whereas a red cue (cue^−^) was unrewarded. Using a 16-channel microwire array, we analyzed pooled channel-level multiunit activity (MUA) aligned to task events. Across sessions, cue^+^ trials showed a learning-related redistribution of event-locked modulation: outcome-locked activity was more prominent early in training, while cue-locked modulation became stronger as performance stabilized, consistent with a temporal-difference–like shift of prediction-related signals. Cue^−^ trials were sparse after early learning and showed limited cue-locked modulation in the available dataset. Together, these results provide initial evidence that pigeon VTA pooled MUA exhibits learning-related dynamics consistent with RPE-like processing and support cross-species comparisons of dopaminergic learning signals.

## Significance Statement

This study provides initial evidence that neurons in the pigeon ventral tegmental area (VTA) may encode reward prediction error (RPE) signals during reinforcement learning. The results show that neural activity related to reward gradually shifts toward the predictive cue as learning progresses, consistent with established models in mammals. These findings suggest that the basic neural processes underlying reward-based learning may be shared across vertebrate species and contribute to a broader understanding of comparative learning mechanisms.

## Introduction

Pioneering work by Schultz and colleagues first demonstrated reward-related phasic activity in midbrain dopamine neurons in nonhuman primates (Schultz et al., 1993). This seminal research paved the way for the influential reward prediction error (RPE) hypothesis, formally proposed in 1997, which posited that dopaminergic neuronal firing encodes the discrepancy between actual received rewards and their prediction (Schultz et al., 1997). Specifically, neuronal activity is potentiated when outcomes exceed expectations (positive RPE) and suppressed when outcomes fall short (negative RPE). Such RPE signals are considered crucial for updating behavioral policies during reinforcement learning: positive errors reinforce preceding actions, while negative errors drive behavioral adjustments, thereby optimizing future reward acquisition (Schultz et al., 1997; Kasdin et al., 2025). Converging evidence has robustly established dopaminergic neurons within the ventral tegmental area (VTA) as a primary neural substrate for these RPE computations, laying the neurobiological groundwork for understanding reward-guided learning and decision-making (Romo and Schultz, 1990; Schultz et al., 1993, 1997; Montague et al., 1996).

Since the introduction of the RPE theory by Schultz, the neural encoding of RPEs has been extensively investigated and robustly validated across diverse mammalian species. Studies employing electrophysiological and electrochemical techniques have demonstrated that multiple brain regions—including the VTA, nucleus accumbens, and ventral striatum—are involved in encoding RPE signals in both nonhuman primates (Stauffer et al., 2014; Basanisi et al., 2023) and rodents (Pan et al., 2005; Flagel et al., 2011; Takahashi et al., 2016; Goedhoop et al., 2023). These signals are critical for guiding reward-based learning and modulating motivational states. Research in humans has provided converging lines of evidence. In humans, dopaminergic activity is typically inferred using functional magnetic resonance imaging (fMRI), where changes in blood oxygenation level-dependent signals have been shown to reflect RPE-related neural activity (D’Ardenne et al., 2008; Daw et al., 2011). Complementing this indirect approach, fast-scan cyclic voltammetry has enabled real-time measurement of subsecond dopamine fluctuations in specific brain regions, providing direct evidence that human dopaminergic systems encode RPEs (Kishida et al., 2016; Sands et al., 2023).

Research on RPE signals has also encompassed avian species, with a predominant focus on songbirds. Song learning in songbirds exhibits complexity, which shows intriguing parallels with human language acquisition. Previous studies have demonstrated that dopaminergic neurons in songbirds generate RPE signals based on auditory feedback from vocal performance, thereby reinforcing or suppressing specific song patterns (Kubikova and Kostál, 2010; Gadagkar et al., 2016; Chen and Goldberg, 2020; Toutounji et al., 2024). In contrast to the auditory-driven learning mechanisms of songbirds, pigeons exhibit strong visual acuity and advanced cognitive capabilities (Usherwood et al., 2011; Clayton and Emery, 2015), making them a valuable model for investigating visually guided reinforcement learning and RPE coding. As nonsongbirds, pigeons diverge significantly from both mammals and songbirds in terms of brain architecture (Carrillo and Doupe, 2004; Von Eugen et al., 2020), and the functional organization of the VTA–basal ganglia circuitry remains poorly characterized.

Previous study has identified RPE signals in the nidopallium caudolaterale (NCL) of pigeons, which shift temporally from the moment of reward delivery to the onset of the cue stimulus (Packheiser et al., 2021). This temporal shift aligns with the predictions of the temporal difference learning model. However, the VTA, which contains a high concentration of dopaminergic neurons and is widely recognized as the principal source of RPE signals, has not yet been definitively shown to encode RPE signals in pigeons in a manner comparable to mammals. Furthermore, how the VTA dynamically modulates RPE signals across trials in pigeons remains poorly understood.

As research into reinforcement learning has advanced, it has become increasingly evident that RPE signaling in the brain is a dynamic process characterized by temporal shifts over the course of learning. Extensive studies in mammals have demonstrated that, as learning progresses, RPE signals gradually shift from the time of actual reward delivery to the onset of conditioned stimuli that predict the reward (Day et al., 2007; Enomoto et al., 2011; Clark et al., 2013; Schultz, 2016; Zhong et al., 2017). This temporal migration reflects a fundamental mechanism by which the brain dynamically updates reward expectations and optimizes behavior. However, studies on the temporal dynamics of RPE signals in nonmammalian species such as birds remain limited, particularly in the context of operant conditioning and complex learning tasks, where such research is relatively scarce.

To illustrate the neural coding properties of the pigeon VTA during reinforcement learning, we employed an operant conditioning paradigm utilizing visual cues. By integrating channel-level multiunit activity (MUA) recordings with behavioral analysis, we examined VTA activity as pigeons engaged in decision-making processes. Our research contributes to provide insights into the shared and distinct mechanisms of reinforcement learning between avian and mammalian species.

To our knowledge, this is the first study to provide direct electrophysiological evidence that the pigeon VTA encodes RPE signals, thereby extending cross-species comparative research on reinforcement learning.

## Materials and Methods

### Animals

Three healthy adult pigeons (*Columba livia*; P109, female; P117, female; and P121, male; 470–550 g) were used. Pigeons were housed in a well-ventilated aviary (3 × 3 × 2 m) with *ad libitum* access to water under standard laboratory conditions. To maintain task motivation, pigeons were food-restricted during the training/recording period, while body weight and health status were monitored and maintained within normal ranges. Although the number of subjects was limited, each pigeon completed repeated sessions with many trials, yielding a total of 455 valid trials across individuals after quality control. The study was conducted according to the guidelines of the Declaration of Helsinki and approved by the Life Science Ethical Review Committee of Zhengzhou University (No. ZZUIRB2022-44).

### Experimental paradigm

The experiment began with an operant pretraining phase in which pigeons were trained to peck a key to obtain a food reward, thereby establishing an association between specific actions and reward outcomes. Learning performance was monitored throughout this phase, and individuals demonstrating robust acquisition were selected for subsequent testing.

**Figure 1.**
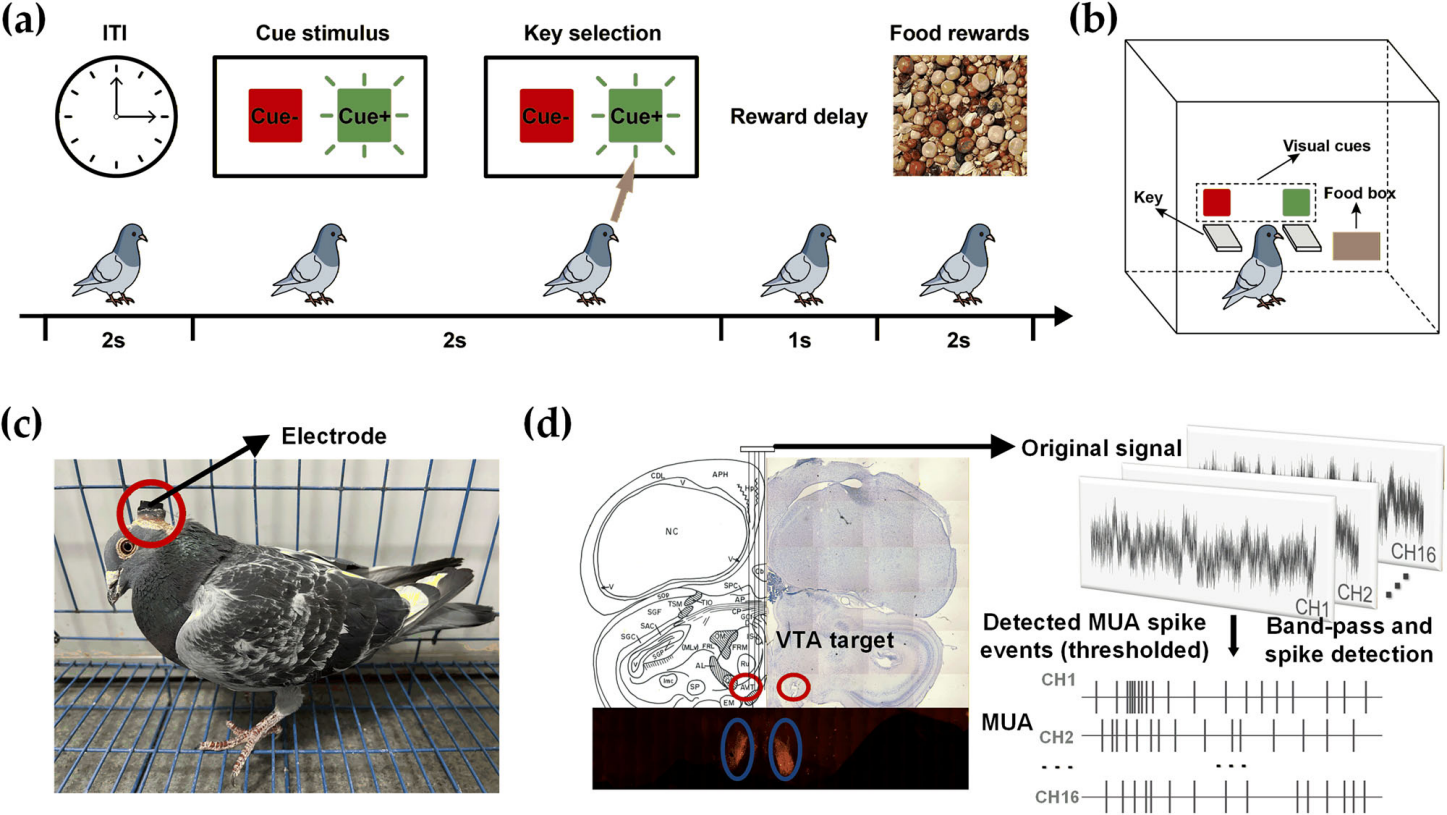
Task design, recording setup, and VTA electrode localization. ***a***, Schematic of the reinforcement learning experimental paradigm. In each trial, either a green (cue^+^) or red (cue^−^) LED light is randomly illuminated for 2 s. If the pigeon pecks the green key during this period, it receives a 2 s food reward, followed by a 2 s intertrial interval. Pecks on the red key or no response result in no reward and immediate transition to the next trial. For neural analyses, precue/cue/outcome epochs were defined as 0.5 s windows aligned to cue onset and outcome time. ***b***, A diagram of the experimental apparatus. ***c***, Pigeon with implanted electrode. ***d***, Electrode implantation site, histological verification, raw traces and detected MUA spike events from the 16 recording channels.

In the formal reinforcement learning task, two pecking keys were presented, each paired with a specific visual cue—one with green light and the other with red. When a green light appeared on a key, a pecking response from the pigeon resulted in a food reward. In contrast, when a red light was displayed, pecking the key yielded no reward. The association between visual cues (green, cue^+^; red, cue^−^) and reward outcomes was fixed across all pigeons, while the spatial positions of the two cues on the left and right keys were randomized on each trial to avoid side bias. This design allowed pigeons to learn the associations between visual stimuli (light cue), behavioral responses (key pecking), and outcomes (food). Neural activity in the VTA was recorded throughout the task. A schematic of the experimental paradigm is shown in Figure 1*a*.

The reinforcement learning procedure was as follows. Each pigeon was first placed inside the experimental apparatus (Fig. 1*b*) for a brief acclimation period to ensure environmental adaptation and behavioral stabilization. At the start of each trial, one of two LEDs—green (cue^+^) or red (cue^−^)—was randomly illuminated for 2 s. If the pigeon pecked the green key within this time window, the food box was opened, delivering a food reward for 2 s, followed by a 2 s intertrial interval. In contrast, if the pigeon pecked the red key or made no response, no reward was given, and the trial transitioned directly into the intertrial interval. Each session was preset to include 100 trials, encompassing all three possible behavioral outcomes: pecking the green key, pecking the red key, or failing to respond.

All experimental sessions were conducted daily between 3 and 4 P.M., with each session typically lasting ∼25–30 min. This ensured a continuous learning process without fatigue. A pigeon was considered to have acquired the target behavior pattern once its correct response rate to the green key exceeded 85% within a single session.

### Surgery

After pretraining, pigeons underwent electrode implantation surgery. Animals were anesthetized via intramuscular injection of 2% sodium pentobarbital (0.2 ml per 100 g body weight) and positioned in a customized stereotaxic apparatus. A 16-channel tungsten microwire array [Kedou (Suzhou) Brain Computer Technology; wire diameter 50 μm; interelectrode spacing 300 μm] was chronically implanted targeting the VTA (Fig. 1*c*,*d*). Implantation coordinates were AP 4.25 ± 0.2 mm, ML 1.5 ± 0.2 mm, and DV 9.50 mm. The array remained in a fixed position throughout all behavioral sessions (no microdrive advancement).

### Neurophysiological data acquisition and preprocessing

After postoperative recovery, pigeons performed the reinforcement learning task while neural and behavioral data were recorded simultaneously. Neural activity was amplified and recorded with a multichannel acquisition system (Cerebus, 128 channels, Blackrock Microsystems) at 30 kHz. Raw signals were bandpass filtered (0.25–5 kHz; Butterworth) to isolate spiking activity.

**Figure 2.**
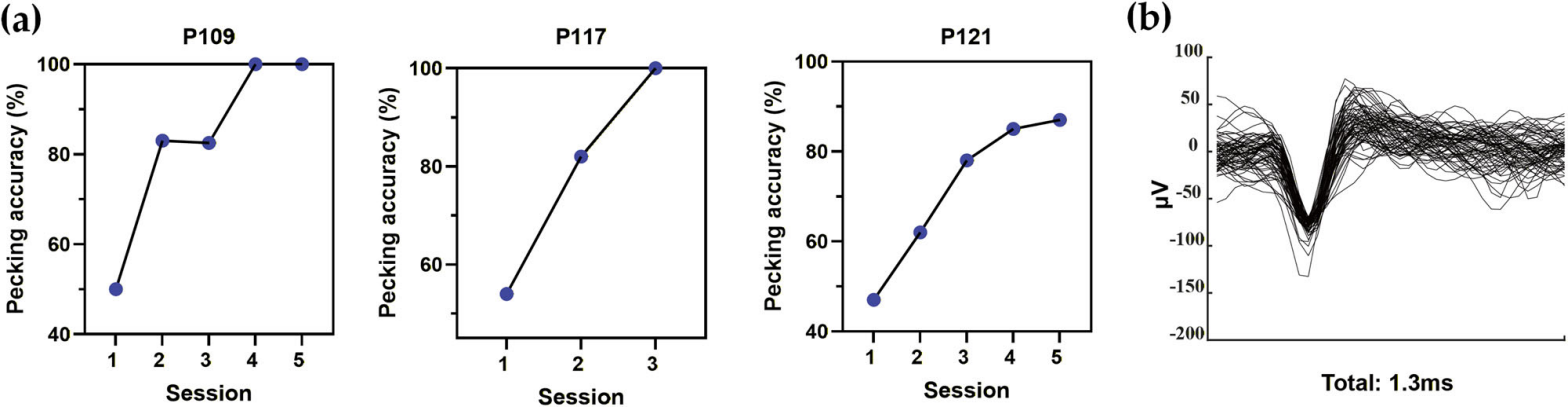
Behavioral learning curves and representative VTA spike waveforms. ***a***, Key pecking accuracy of P109, P117, and P121 across sessions. ***b***, Spike waveform samples after preprocessing.

Spike events were detected independently on each channel using a fixed threshold set to 5× the noise standard deviation. For each detected event, a 1.3 ms waveform segment (39 samples at 30 kHz) was extracted and aligned to the negative peak to reduce temporal jitter (Li et al., 2023). Importantly, given the 300 μm interelectrode spacing and the study's focus on population-level dynamics, spiking signals were treated as channel-level MUA rather than well-isolated single units. Therefore, analyses were performed using channel-level spike times (MUA events) without making single-unit claims. Example preprocessed waveforms are shown in Figure 2*a*.

### Histological verification

Following the completion of electrophysiological recordings, pigeons were deeply anesthetized, and electrolytic lesions were made at the electrode implantation sites by applying direct current (1.1 mA for 30 s, repeated three times) to mark the recording locations. Cardiac perfusion was then performed sequentially with physiological saline followed by 4% paraformaldehyde to fix the tissue. The brain was extracted and postfixed in 4% paraformaldehyde for an additional 16 h and then cryoprotected in 30% sucrose solution until fully dehydrated. After cryoprotection, the brain tissue was frozen and coronally sectioned at a thickness of 40 μm. Brain sections were processed with Nissl staining (Cresyl violet) and immunofluorescence staining (tyrosine hydroxylase), respectively. Stained sections were compared with a standard pigeon brain atlas to verify the accuracy of the electrode implantation sites.

### Behavioral data analysis

Behavioral performance was quantified for each pigeon and each session. Pecking accuracy was defined as the proportion of green-key selections among all key-peck trials (green + red) within a session. Session-by-session changes in pecking accuracy are shown in Results (Fig. 2*a*). Overall, pigeons progressively increased their preference for the green key across training, consistent with learning the association between cue, key selection, and reward outcome.

### MUA data analysis

Spike activity was analyzed based on channel-level MUA recorded from a 16-channel microwire array. For each session, the continuous signal from each channel was bandpass filtered (250–5,000 Hz), and spike events were detected using a fixed threshold of 5× the noise standard deviation. Spike times from all 16 channels were pooled to characterize population-level VTA spiking dynamics.

#### Trial-level quality control

To ensure data quality, we excluded (1) premature responses (response time <500 ms from cue onset) and (2) trials contaminated by large-amplitude wing flapping or other movement-related artifacts. Unless otherwise stated, all analyses were conducted on the remaining valid trials.

#### Spike counts and rate metrics

To quantify how spiking was distributed across task epochs, we computed an epoch-wise spike proportion from pooled VTA MUA. For each valid trial, spike events detected across the 16 channels were pooled and counted within three predefined 0.5 s epochs: precue reference (−0.5 to 0 s relative to cue onset), cue (0–0.5 s), and reward/outcome (0–0.5 s relative to reward delivery; rewarded trials only). Let $N_{\mathrm{pre}}$, $N_{\mathrm{cue}}$, and $N_{\mathrm{rew}}$ denote the pooled spike counts within these epochs. For cue^+^ (rewarded) trials, we defined the proportion of spikes in each epoch as follows:

$$p_{\mathrm{pre}}=\frac{N_{\mathrm{pre}}}{N_{\mathrm{pre}}+N_{\mathrm{cue}}+N_{\mathrm{rew}}},\;\;p_{\mathrm{cue}}=\frac{N_{\mathrm{cue}}}{N_{\mathrm{pre}}+N_{\mathrm{cue}}+N_{\mathrm{rew}}},\;p_{\mathrm{rew}}=\frac{N_{\mathrm{rew}}}{N_{\mathrm{pre}}+N_{\mathrm{cue}}+N_{\mathrm{rew}}},$$

where p∈[0,1] represents the fraction of spikes occurring during precue relative to the total spikes across the three epochs in that trial.

For cue^−^ (nonrewarded) trials, because reward delivery does not occur, analyses were restricted to the precue, and cue epochs and proportions were computed as follows:

$$p_{\mathrm{pre}}=\frac{N_{\mathrm{pre}}}{N_{\mathrm{pre}}+N_{\mathrm{cue}}},\;\;p_{\mathrm{cue}}=\frac{N_{\mathrm{cue}}}{N_{\mathrm{pre}}+N_{\mathrm{cue}}}.$$

**Figure 3.**
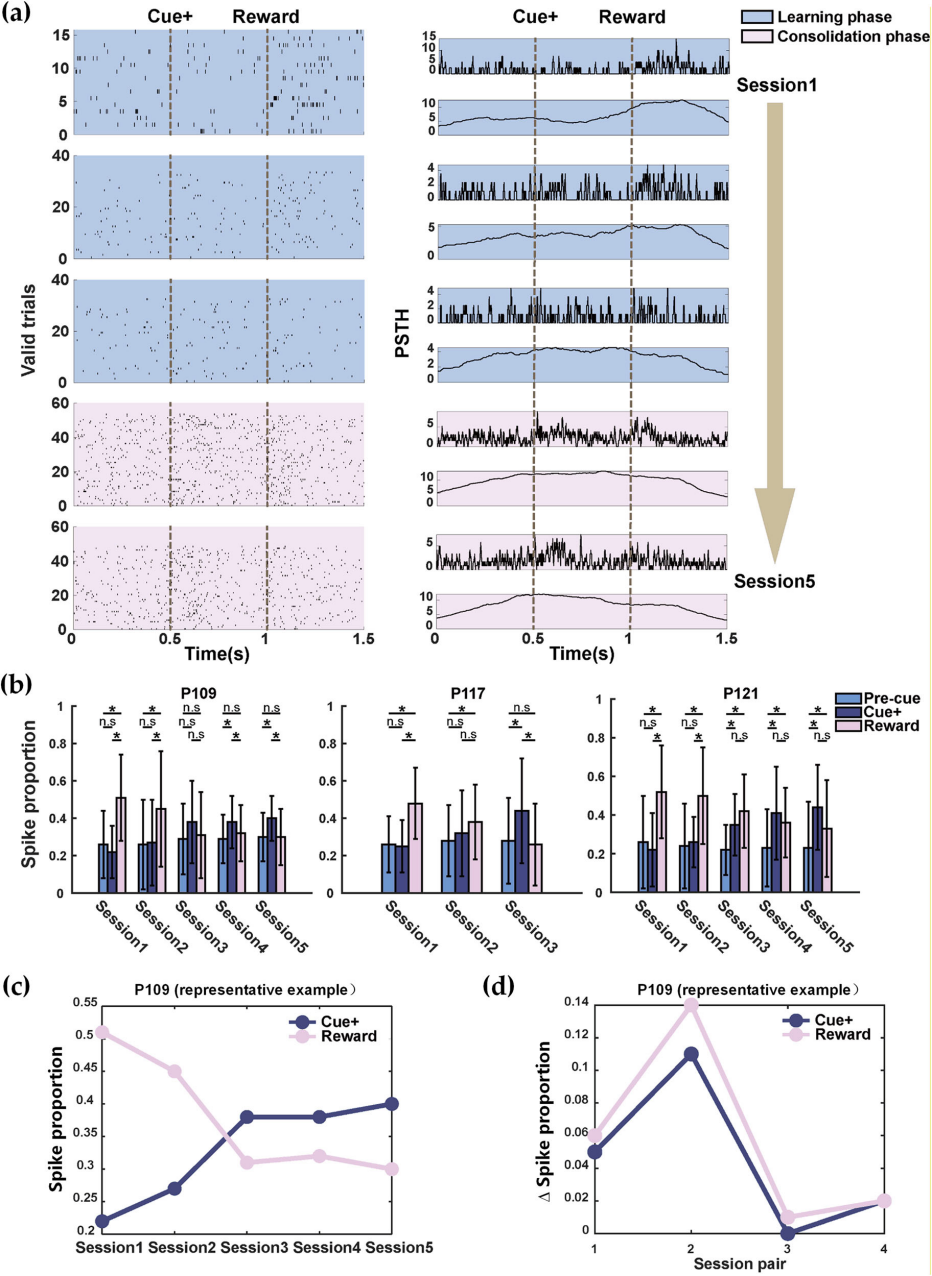
Learning-related redistribution of VTA pooled MUA across task epochs during cue^+^ trials. ***a***, Example cue^+^ rasters (left) and PSTHs (right) for pigeon P109 across sessions, aligned to cue onset (0.5 s) and reward delivery (1.0 s; dashed lines). Background shading indicates sessions assigned to the learning phase (blue) versus the consolidation phase (pink) based on behavioral performance. For each session, PSTHs are shown at two temporal resolutions (upper, 10 ms bins; lower, 100 ms bins with a 5-point moving average for visualization). ***b***, Session-wise spike proportions for cue^+^ trials in the precue reference, cue^+^, and reward epochs for each pigeon (mean ± SD across valid trials). Statistical significance is indicated above bars (see Materials and Methods, Statistical analysis). ***c***, Representative example from pigeon P109 illustrating how the session-wise mean spike proportion evolves across training. Each point corresponds to the same mean value shown for P109 in panel ***b*** (computed across valid trials within that session for the cue and reward epochs). The curve is provided as a visualization of the learning-related trajectory rather than a group-level summary. ***d***, Session-to-session change in the same metric for P109, computed as Δ = value (Session *n* + 1) − value (Session *n*), where each value is the session-wise mean spike proportion (identical to the corresponding bar height in panel ***b***).

**Figure 4.**
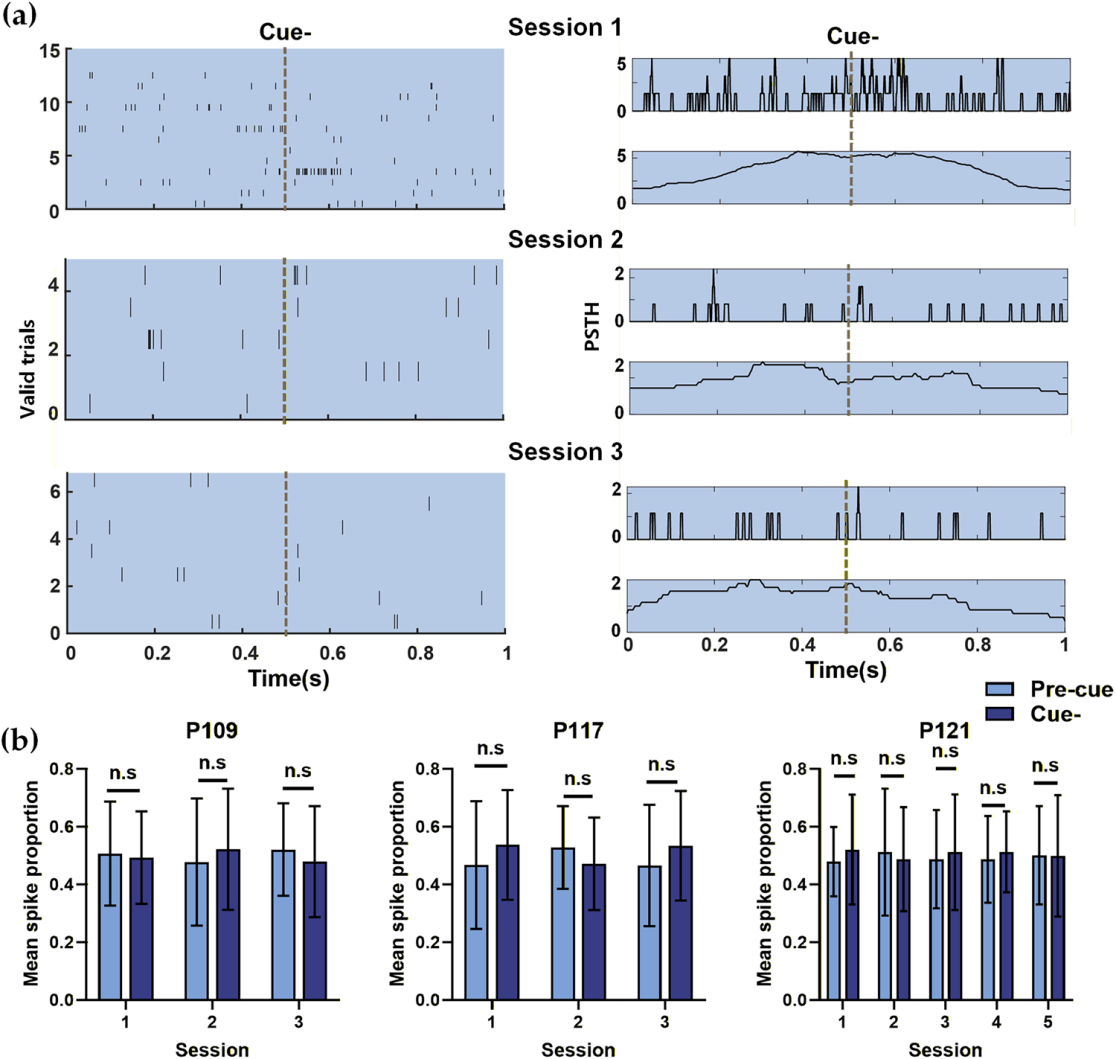
Cue^−^ trials show limited cue-locked modulation in VTA pooled MUA. ***a***, Example cue^−^ rasters (left) and PSTHs (right) for pigeon P109 across Sessions 1–3, aligned to cue− onset (dashed line). The *y*-axis indexes valid trials after trial-level quality control. For each session, PSTHs are shown at two temporal resolutions (upper, 10 ms bins; lower, 100 ms bins with a 5-point moving average for visualization). ***b***, Session-wise comparison of cue^−^ spike proportions between the precue reference and cue^−^ epochs for each pigeon (mean ± SD across valid trials). “n.s.” indicates no significant difference between epochs (see Materials and Methods, Statistical analysis).

These proportion metrics were computed on a trial-by-trial basis and then summarized at the session level by averaging across valid trials (mean ± SD), as reported in Figures 3 and 4.

#### Raster plots, PSTHs, and sliding-window analysis

To visualize task-related spiking, we constructed raster plots and peristimulus time histograms (PSTHs) based on pooled MUA. In raster plots, the *y*-axis indicates the index of valid trials, and each tick represents a detected MUA spike event within that trial (pooled across all 16 channels). PSTHs were generated by binning spike times using a fixed bin width, computing spike counts per bin for each trial, converting counts to firing rates, and then averaging across valid trials. PSTHs are reported as either pooled-array activity (spikes/s) or channel-normalized activity (spikes/s/channel), as specified in the corresponding figure captions.

To quantify temporal dynamics in spiking strength, we performed a sliding-window analysis on spike counts derived from the pooled MUA. Spike counts were computed within a moving window (window size and step as specified in the figure caption), converted to firing rates, and used to generate continuous firing-rate curves over time. For a given epoch, the area under the firing-rate curve provides a compact measure of response magnitude within that temporal segment.

### Trial exclusion criteria, sample-size summary, and analysis windows

Trials in which pigeons pecked within 0.5 s of light onset were classified as premature responses and excluded from analysis. Trials contaminated by large-amplitude wing flapping or other movement-related artifacts were also excluded. These exclusion criteria were applied consistently across pigeons and sessions; however, the proportion of excluded trials varied across individuals due to differences in behavior and movement.

After trial-level quality control, a total of 451 valid trials were retained across the dataset (summarized by pigeon in Table 1 and by session in Table 2). All sessions were recorded using the same 16-channel array, and spike events were analyzed as pooled MUA across channels.

**Table 1.** Summary of trial inclusion/exclusion and recording channels (pooled MUA) by pigeon

Pigeon ID (sex)	Total trials/valid trials/ excluded trials	Exclusion reasons (trial count)	Number of channels
P109 (female)	267/210/57	Premature response (<500 ms; *n* = 35) large-amplitude wing flapping (*n* = 22)	16
P117 (female)	153/89/64	Premature response (<500 ms; *n* = 33) large-amplitude wing flapping (*n* = 31)	16
P121 (male)	319/152/167	Premature response (<500 ms; *n* = 97) large-amplitude wing flapping (*n* = 70)	16

**Table 2.** Session-wise summary of trial counts used for pooled MUA analyses

Pigeon ID	Session	Extracted key-peck trials	Valid trials	Channels included (*n*)
P109	S1	41	28	16
	S2	55	39	16
	S3	53	40	16
	S4	61	54	16
	S5	57	49	16
P117	S1	45	21	16
	S2	57	35	16
	S3	51	33	16
P121	S1	63	26	16
	S2	72	33	16
	S3	68	34	16
	S4	67	32	16
	S5	49	27	16

To examine learning-related dynamics, sessions were categorized into a learning phase and a consolidation phase based on behavioral performance, using a correct response rate threshold of 85% to delineate the boundary. The learning phase included sessions before the animal achieved ≥85% correct responses, while the consolidation phase included sessions once performance met or exceeded this threshold. We note that this 85% threshold was used as an operational criterion for phase labeling (learning vs consolidation), rather than as a strict stopping rule. Accordingly, the number of recorded sessions differed across pigeons: P109 and P121 include additional postacquisition sessions to sample stable (consolidation) activity, whereas P117 reached criterion by Session 3 and therefore did not undergo further training/recording sessions.

For event-aligned neural analyses, the precue period was defined as the 0.5 s precue reference window immediately preceding cue onset (−0.5 to 0 s), the cue period as the 0.5 s window immediately following cue onset (0–0.5 s), and the reward/outcome period as the 0.5 s window following reward delivery (0–0.5 s relative to reward onset; rewarded trials only). This precue definition was chosen to quantify firing changes in a temporally contiguous window around cue onset; we note that as learning progresses, the precue window may include anticipatory activity and therefore precue referenced effects are interpreted conservatively. For visualization, these three event-locked segments were concatenated into a 1.5 s analysis window (precue to cue to outcome), with time shown relative to cue onset (and outcome onset where applicable).

### Statistical analysis

Statistical analyses were performed using nonparametric tests. For paired comparisons within the same trials or sessions (e.g., precue vs cue or precue vs reward within a session), we used the Wilcoxon signed-rank test. For comparisons between independent groups (e.g., learning vs consolidation phase when treated as independent samples), we used the Wilcoxon rank-sum test. All tests were two-tailed with significance set at p<0.05. In figures, * indicates p<0.05and n.s. indicates nonsignificance.

## Results

We first summarize the behavioral learning progress and provide an overview of the recorded spiking signals. Across pigeons, cue^+^ (green-key) pecking accuracy increased over training sessions, reaching a stable high level as the task was acquired (Fig. 2*a*; P109 and P121, Sessions 1–5; P117, Sessions 1–3). To illustrate the quality of the recorded spiking activity after preprocessing, we also show representative spike waveform samples extracted from the VTA recordings (Fig. 2*b*). Building on this behavioral improvement and signal overview, we next analyzed event-aligned VTA pooled MUA dynamics during cue^+^ trials across sessions.

### Learning-related temporal redistribution of VTA pooled MUA during cue^+^ trials

To examine how event-aligned VTA activity evolves across learning in cue^+^ trials, we analyzed pooled channel-level MUA across three predefined epochs (precue reference, cue, and reward) and tracked changes over training sessions (Fig. 3). In pigeon P109 (five sessions total), Sessions 1–3 were classified as the learning phase and Sessions 4–5 as the consolidation phase based on behavioral performance. The raster plots and PSTHs illustrate that, during early learning (Session 1), event-locked modulation was more concentrated around the reward period, whereas cue-locked modulation was relatively weak (Fig. 3*a*). As training progressed (Sessions 2–3), cue-aligned modulation became more apparent while reward-aligned modulation diminished. In the consolidation phase (Sessions 4–5), modulation was predominantly aligned to the cue period, consistent with a redistribution of event-locked activity from outcome to cue as performance stabilized.

We quantified this pattern using trial-level spike proportions computed on valid trials after quality control. Across pigeons, session-wise summaries show that the reward-epoch proportion was highest early and decreased with learning, whereas the cue-epoch proportion increased and became larger in later sessions (Fig. 3*b*). As a representative example, the corresponding session-wise trajectories and session-to-session changes for pigeon P109 are shown in Figure 3, *c* and *d*. Within each pigeon and session, session-level mean spike proportions differed significantly across the precue, cue^+^, and reward epochs in multiple sessions (Fig. 3*b*; see Materials and Methods, Statistical analysis). Together, these results indicate that cue^+^ learning is accompanied by a robust temporal redistribution of VTA pooled spiking from outcome-locked to cue-locked epochs, consistent with a learning-related shift from outcome-locked to cue-locked modulation.

### Early rapid changes followed by stabilization in cue^−^ and reward-locked activity

To summarize the time course of learning-related modulation, we focused on the representative pigeon P109 and examined how cue^−^ and reward-epoch spike proportions evolved across sessions. In P109, the cue-epoch proportion increased, and the reward-epoch proportion decreased primarily during the early sessions (Sessions 1–3), whereas both measures showed only modest variation once performance stabilized (Sessions 4–5; Fig. 3*c*). Consistent with this, the session-to-session change (Δ proportion; Session *n* + 1 − Session *n*) was largest early and became small in later session pairs (Fig. 3*d*). This pattern parallels the behavioral learning curve, indicating that the redistribution of VTA event-locked activity occurs most prominently during early training and stabilizes as the task is acquired. Thus, both behavior and neural measures converge on a fast initial learning phase followed by a plateau, during which cue-locked modulation is maintained with reduced session-to-session change.

### Cue^−^ trials show limited cue-locked modulation

To evaluate cue-locked modulation during cue^−^ (red-key) choices, we analyzed pooled VTA MUA on trials in which pigeons selected the red key, focusing on the precue reference and cue epochs (Fig. 4). In the illustrative example P109, raster plots and PSTHs aligned to cue^−^ onset showed broadly similar spiking density before and after cue presentation across Sessions 1–3 (Fig. 4*a*). Consistent with this qualitative pattern, session-wise trial–level comparisons between the precue and cue windows did not reveal a significant difference in mean spike proportion for cue^−^ trials (Fig. 4*b*; n.s. across sessions for each pigeon). Together, these results indicate that cue^−^ trials exhibited limited cue-locked modulation in the available dataset.

## Discussion

This study combined behavioral analysis and VTA recordings to examine learning-related changes in event-aligned VTA activity in pigeons. Using pooled channel-level MUA (spike events pooled across the 16 recording channels), we observed a learning-related redistribution of event-locked modulation in cue^+^ trials, with outcome-locked activity being more prominent early in training and cue-locked modulation becoming more apparent as performance stabilized. These patterns are consistent with a temporal-difference–like account of RPE signaling. Cue^−^ trials were sparse after early learning; therefore, cue^−^ activity is reported descriptively and we do not draw strong conclusions regarding value-dependent temporal effects from those analyses. Overall, our results suggest parallels between avian VTA activity and RPE-like dynamics reported in other species, while emphasizing the exploratory nature of the present dataset.

In cue^+^ trials, pooled VTA MUA showed a learning-related temporal redistribution from outcome-locked to cue-locked modulation. This dynamic is consistent with predictions from the temporal difference learning model (Sutton and Barto, 2018). According to the temporal difference model, RPE signals are expected to move in time from the reward to the predictive cue as learning advances, representing the increasing precision of reward prediction in reinforcement learning. Our findings provide further empirical support for this theoretical framework and reveal that the rate of signal shift is faster during the early stages of learning and gradually stabilizes over time. This shift may reflect the VTA's role in rapidly updating prediction errors during initial learning phases, with the magnitude of signal adjustment decreasing as reward expectations become more precise (Lak et al., 2016).

Pigeon VTA pooled channel-level MUA exhibited RPE-like, event-locked dynamics and a learning-related temporal redistribution from outcome-locked to cue-locked modulation in cue^+^ trials. This pattern is consistent with cross-species reports of TD-like temporal redistribution in dopaminergic systems in mammals (Romo and Schultz, 1990; Schultz et al., 1997; Niv, 2009; Kim et al., 2020; Schultz, 2024), suggesting that similar computational principles may operate across species.

Cue^−^ (red-key) trials were sparse after early learning; therefore, cue^−^ activity is reported descriptively, and we refrain from making claims about value-dependent temporal shifts. Although prior studies suggest dopaminergic signals can be sensitive to reward value (Tobler et al., 2005; Rios et al., 2023), our dataset is not sufficient to evaluate such effects reliably.

Nevertheless, this study has several limitations that warrant discussion and further investigation. The first limitation of this study is the small sample size (*N* = 3 pigeons). While our findings provide preliminary evidence for the dynamic encoding of RPE signals in the pigeon VTA, the limited cohort inevitably reduces the statistical power of our analyses and constrains the generalizability of the results. However, it should be emphasized that avian neurophysiology research often adopts small cohorts combined with intensive within-subject trial repetitions. In our study, each pigeon contributed hundreds of valid trials, yielding a total of 451 trials across individuals, and the behavioral and neural patterns were highly consistent across subjects. This design provides sufficient reliability for exploratory work and establishes a solid foundation for future studies, which will need to expand the sample size to validate and extend these observations.

Second, this study primarily focused on the signal activity of the VTA as a single brain region, without systematically examining its interactions with other brain regions. Existing research has shown that the activity of NCL dopaminergic neurons is strongly influenced by the VTA (Kalt et al., 1999; Von Eugen et al., 2020), and its role in reward learning may be highly correlated with the VTA. Future research could further elucidate the role of the VTA–NCL network in RPE signal generation through multibrain region joint recordings.

Finally, another limitation concerns the task design. This study did not include reward omission or extinction (forgetting) paradigms. According to the classical RPE theory, in contrast to positive RPE, the activity of dopamine neurons decreases below precue levels when the expected reward is omitted (Tian and Uchida, 2015). Similarly, extinction tasks, in which a previously learned association is no longer reinforced (Packheiser et al., 2019), are critical for dissociating genuine RPE signals from neural responses that simply reflect stable stimulus–reward associations. Our deterministic reinforcement schedule was deliberately chosen to establish a clear precue of VTA activity in pigeons, enabling us to identify RPE-like dynamics under controlled conditions. Future research should therefore incorporate probabilistic reward paradigms, explicit reward omission, and extinction experiments to systematically differentiate RPE signals from other neural representations and to further validate the generalizability of our findings.

## Synthesis

Reviewing Editor: Timothy Jarome, Virginia Polytechnic Institute and State University Decisions are customarily a result of the Reviewing Editor and the peer reviewers coming together and discussing their recommendations until a consensus is reached. When revisions are invited, a fact-based synthesis statement explaining their decision and outlining what is needed to prepare a revision will be listed below. The following reviewer(s) agreed to reveal their identity: Kaiser Arndt, Roland Pusch.

Both reviewers found the work to be interesting and of importance to field. However, several major concerns were noted that need to be addressed.

1. There are several concerns about details regarding the electrode device and spike sorting.

a. First, more details are needed about the microelectrode array, such as provider or custom construction and inter channel distances. This is vital as successful spike sorting of neurons requires close electrodes to be able to disambiguate clustered action potentials. This information will also affect the claim of 'units' since if the electrodes are far apart (greater than 50 um) they could be classified as multiunit activity. In the method section it should be stated what criteria must be met to qualify recordings as single- or multi-units. Were the electrodes mounted on a microdrive and moved between the different behavioral sessions? Or were they chronically implanted without further movement? What kind of geometrical electrode design was used?

b. Please include a figure and more detailed description of the number of units recorded per session, and how they were spike sorted into and preprocessed into individual units. Figure 1d shows the filtered spike train on the 16 consecutive channels. Here, each channel carries spikes that are clearly different between the channels. This implies that here single/multi-unit activity was recorded. Of the 13 sessions reported here, 455 valid trials have been extracted. This means that 208 (13 session x 16 channels) single units are possible. Table 1 should additionally contain the number of single-units and multi-units recorded during the valid trials for each pigeon. This information is also important when reporting the statistical analysis. By convention, at least ~100 single units should be included in the data set.

c. It is unclear how the mean spike firing rates were calculated. Was this calculation based on a single cell? Please include a more detailed description on how you process the data to identify units. Did you sort units on a trial by trial basis or the entire recording session?

d. How long was each recording session or length of data used to spike sort? This will again effect spike sorting outcomes as successful spike sorting needs a minimum of 30 minutes of recording with the best electrodes. If less time than this, the sorting quality decrements quickly and could be considered multiunit activity (MUA).

2. Section 3.1 "behavioral data analysis" should be moved up the methods section.

3. What is the time frame between sessions, 1 day, multiple days, and months? It would be nice to expand description to account for this. It provides insight into the larger learning timeline these animals are experiencing

4. Please format the legends of figures to be more distinguishable from the text. For further revisions.

5. Figure 2b and Table 2 should also include the other birds. Only three birds have been used in this study, and adding this information will neither overcrowd the figure nor the table. Additionally, the omissions and the removed trials should be given to have an estimate for the session quality.

6. Figure 2c and figure 3b contain partly overlapping information. We suggest to remove figure 2c, because the information is redundant.2. Expanding figure 4 to resemble figure 3 would also be nice. Was there a "timeout" period after selection of the red key that could show comparison to the reward period during successful trials?

7. Figure 3: It is unclear what is shown in the figure and what information is contained in the respective subpanels:

Figure 3a: It is assumed that the figure shows one single-unit for each session of Pigeon 109. Are these different single units? Or is it the same cell? Based on Table 2, Session 5 should entail 60 green key pecking trials. However, no spiking activity is visible beyond trial ~45. How is this possible.

The binning is very fine and the PSTH might improve when the bin size is slightly increased.

The time windows used for the analysis should not be presented as continuous curve, because it depends on the behavior of the animal in the specific trial. This should be clearly mentioned in the text, and it should be visible in the figure and mentioned in the figure caption. Additionally, the spike-density function shows a pronounced anticipatory response immediately prior cue onset. It might be useful to define the baseline time window in the middle of the ITI (-1- -0.5s) rather than directly prior cue onset (-0.5 - 0s).

Figure 3b: What is shown here? It is suggested that this is the mean relative firing rate of all neurons for each pigeon for each time window. However, the statistics are not reported properly: It is unclear how many neurons are included. The No medians or means are given, it is unclear what the error bars represent, no test statistics are reported, no degrees of freedom are mentioned. In the Methods section, the authors state that they performed a Wilcoxon rank-sum test. How did they corrected for multiple comparisons? Why are only 3 Sessions shown for Pigeon 117? Unfortunately, without this information, it is difficult to evaluate the quality and robustness of the results. The information has to be included.

Fig. 3d it is not clear what is being shown here and what the important implications of this plot are. Please elaborate the description of what this plot is trying to show and its implications.

8. Please expand the description of Exploration phase vs acquisition phase. It is unclear if the exploration phase is the initial acclimation period, or once the animal had reached a 85% success rate, or if this was just after some time. Making this clearer will help with the overall takeaways of the paper. Also suggest changing the nomenclature of the phases: Exploration phase sounds rather random and might be exchanged by "Learning phase". "Acquisition phase" has a specific meaning in extinction learning and might be misunderstood or confusing. Consolidation phase might be more suitable, because learning has (presumably) terminated.

9. Line 90, Section 2.1. Animals: An ethics statement is missing.

10. Line 123: Were the locations of the color presentations counterbalanced? Please specify.

11. Line 302: 3.4. RPE signals exhibit a value-dependent temporal shift: The analysis of the firing rates during the red key choices does not seem to be meaningful due to the lack of data. How many cells were recorded within each session? The claim of the value-dependent temporal shift should be removed.

12. Line 327: The claim of the value-dependent temporal shift should be removed also in the discussion

13. Line 354-364: The use of three pigeons is sufficient and many studies use only a few animals. This is ethically required and does not pose a statistical problem. The important measure is the number of recorded neurons. Unfortunately, this information is not contained in the manuscript.
